# Chemical and Antimicrobial Evaluation of Supercritical and Conventional *Sideritis scardica* Griseb., Lamiaceae Extracts

**DOI:** 10.3390/molecules17032683

**Published:** 2012-03-05

**Authors:** Vanja Tadić, Dragica Bojović, Ivana Arsić, Sofija Đorđević, Ksenija Aksentijevic, Marko Stamenić, Slobodan Janković

**Affiliations:** 1 Institute for Medicinal Plant Research “Dr Josif Pančić”, Department of Pharmaceutical Research and Development, Tadeuša Košćuška 1, Belgrade 11 000, Serbia; E-Mails: ivana.arsic@dioskuri.rs (I.A.); sdjordjevic@mocbilja.rs (S.Đ.); 2 ICN Montenegro, Mitra Bakića 64, Podgorica 20 000, Montenegro; E-Mail: dragicabiljna@t-com.me; 3 Faculty of Veterinary Medicine, Department of Microbiology and Immunology, Univesity of Belgade, Bulevar oslobođenja 18, Belgrade 11 000, Serbia; E-Mail: pojavica@gmail.com; 4 Department of Organic Chemical Technology, Faculty of Technology and Metallurgy, University of Belgrade Karnegijeva 4, P.O. Box 3503, Belgrade 11 120, Serbia; E-Mail: stamena@tmf.bg.ac.rs; 5 Faculty of Medicine, Department of Pharmacology, University of Kragujevac, Slobodana Markovića 69, Kragujevac 34 000, Serbia; E-Mail: slobodan.jankovic@medf.kg.ac.rs

**Keywords:** *Sideritis scardica* Griseb., Lamiaceae (mountain tea), essential oil, hydrodistillation, supercritical carbon dioxide extracts, solvent extraction, terpenoids, antimicrobial activity, GC and GC-MS analysis

## Abstract

*Sideritis scardica* Griseb., Lamiaceae (ironwort, mountain tea), an endemic plant of the Balkan Peninsula, has been used in traditional medicine in the treatment of antimicrobial infections, gastrointestinal complaints, inflammation and rheumatic disorders. This study reports a comparison between conventional (hydrodistillation HD and solvent extraction SE) and alternative (supercritical carbon dioxide SC CO_2_) extraction methods regarding the qualitative and quantitative composition of the obtained extracts as analyzed by GC and GC-MS techniques and their anitimicrobial activity. Different types of extracts were tested, the essential oil **EO** obtained by HD, **EO-CO_2_** and **AO-CO_2_** obtained by SC CO_2_ at different preasures 10 and 30 MPa, at 40 °C, respectively, and the fractions **A**, **B**, **C** and **D** obtained by successive solvent extraction (SE) **A**: ethanol, **B**: diethyl ether, **C**: ethyl acetate and **D**: *n*-butanol). While **EO** was characterized by the presence of the high percentage of oxygenated monoterpenes and sesquiterpenes (30.01 and 25.54%, respectively), the rest of the investigated samples were the most abundant in fatty acids and their esters and diterpenes (from 16.72 to 71.07% for fatty acids and their esters, and from 23.30 to 72.76%, for diterpenes). Microbial susceptibility tests revealed the strong to moderate activity of all investigated extracts against the tested microorganisms (MIC from 40 to 2,560 μg/mL). Although differences in the chemical compositions determined by GC and GC-MS analysis were established, the displayed antimicrobial activity was similar for the all investigated extracts.

## 1. Introduction

The results of numerous preliminary investigations of plants beloning to the genus *Sideritis* L. have revealed plant-derived compounds of particular pharmacological and nutritional interest. *Sideritis* L. (Lamiaceae) includes approximately 150 species of annual and perennial plants distributed mainly in the Medirerranean region. This genus is devided into two subgenera, *Sideritis* and *Marrubiastrum,* formed by the European and Macaronesian species, respectively. So far, different biological activities of *Sideritis* species have been reported: anti-inflammatory, anti-ulcer, analgesic, antimicrobial and antifungal [1,2,3,4,5], immunomodulating [6], macrophage NOS-2-expression inhibiting [7], and hypoglycemic [4]. Recently, aldose reductase inhibiting activity [8], antiproliferative, anticholinesterase and selective estrogen receptor modulator-like effects and cytotoxic properties have also been reported [9,10,11,12]. The previous studies of *Sideritis* species reported the presence of flavonoid aglycones and glycosides, phenolic acids, di- and triterpenoids, fatty acids, coumarins and iridoid glycosides [2,8,10,12,13,14,15,16]. The composition of various *Sideritis* species essential oils has also been studied exhaustively as well [1].

The genus *Sideritis* is represented in Serbia by only one species, *S. montana* L. [17], but because of its pro-oxidant properties this plant has not been used in traditional medicine [18]. *S. scardica* Griseb. (ironwort, mountain tea) is an endemic plant of the Balkan Peninsula belonging to the *Empedoclea* section. Aerial parts of “mountain tea” are traditionally known for their anti-inflammatory, anti-microbial, anti-bacterial, anti-rheumatic and gastroprotective properties. *S. scardica* is used as a loosening agent in bronchitis and bronchial asthma, against common cold and lung emphysema, as well.

With the current trend towards increasing use of traditional medicines, plant-derived agents have been attracting much interest as natural alternatives to synthetic compounds. Since the Middle Age, essential oils have been widely used in the pharmaceutical, sanitary, cosmetic, agricultural and food industries. In recent years, there has been the considerable interest in essential oils extracted from various medicinal plants with the goal of discovering their multifunctional properties in addition to their classical roles as food additives and/or fragrances. Known properties of essential oils include antibacterial, antifungal, antioxidant and anti-inflammatory activities. The most common method of essential oil isolation is hydrodistillation (HD). Although a very simple process, it suffers of many drawbacks: thermal degradation, hydrolysis and solubilization in water of some compounds that alter the flavor and fragrance profile of many essential oils extracted by this technique. Organic solvent extraction (SE) is often used for isolation of active components from plant material in order to preserve thermolabile and highly volatile compounds, but it requires the use of organic solvents. Recently clean techniques, such as supercritical fluid extraction (SFE) have been developed for extracting not only essential oils. but other active component from complex matrices. SFE is a separation technology that uses a supercritical fluid as the solvent, carbon dioxide (CO_2_) being the main supercritical solvent. Carbon dioxide (critical conditions = 30.9 °C and 73.8 bar) is cheap, environmentally friendly and generally recognized as safe. Supercritical CO_2_ (SC-CO_2_) is attractive because of its high diffusivity and its easily tunable solvent strength. Another advantage is that CO_2_ is gaseous at room temperature and ordinary pressure, which makes the recovery of analytes very simple and provides solvent-free analytes [19]. Besides, SFE using CO_2_ allows the extraction of termally labile or easily oxidized compounds. The main drawback of SC-CO_2_ is its low polarity, a problem that can be overcome by employing polar modifiers (co-solvents) to change the polarity of the supercritical fluid and to increase its solvating power towards the analyte of interest.

Considering the differences between the applied extraction methods, which might reflect primarily in enhanced bioavailability of the active principles and the consequent increase of its therapeutic properties, the aim of this study was to evaluate the chemical profile and antimicrobial properties of conventional and supercritical *S. scardica* Griseb., Lamiaceae extracts, **EO**: essential oil obtained by hydrodistillation, **EO-CO_2_**: low volatile fraction obtained by supercritical carbon dioxide extraction at 10 MPa and 40 °C, **AO-CO_2_**: the fraction obtained by supercritical fluid extraction at higher pressure, 40 MPa and 40 °C, and fractions obtained by successive solvent extraction: samples **A** - ethanol, **B** - diethyl ether, **C** - ethyl acetate and **D** - *n*-butanol extracts. The chemical composition of the investigated extracts was determined by GC and GC-MS analysis. In addition, the *in vitro* antimicrobial activity of the investigated extracts was assessed against Gram-positive and Gram-negative bacteria and yeast strains of medicinal relevance applying the broth microdillution method. As far as a literature survey ascertained, there have been no reports on the chemical composition or antimicrobial activity of SC-CO_2_ extracts isolated from *S. scardica* at 10 MPa and 30 MPa at 40 °C. Papaefstathiou *et al*. reported the successful SFE extraction of added value components from *S. raeseri* [20], however, we have not found in the open literature any report regarding the chemical composition and antimicrobial activity of SC-CO_2_ extracts isolated from *S. scardica*. 

## 2. Results and Discussion

**Figure 1:**
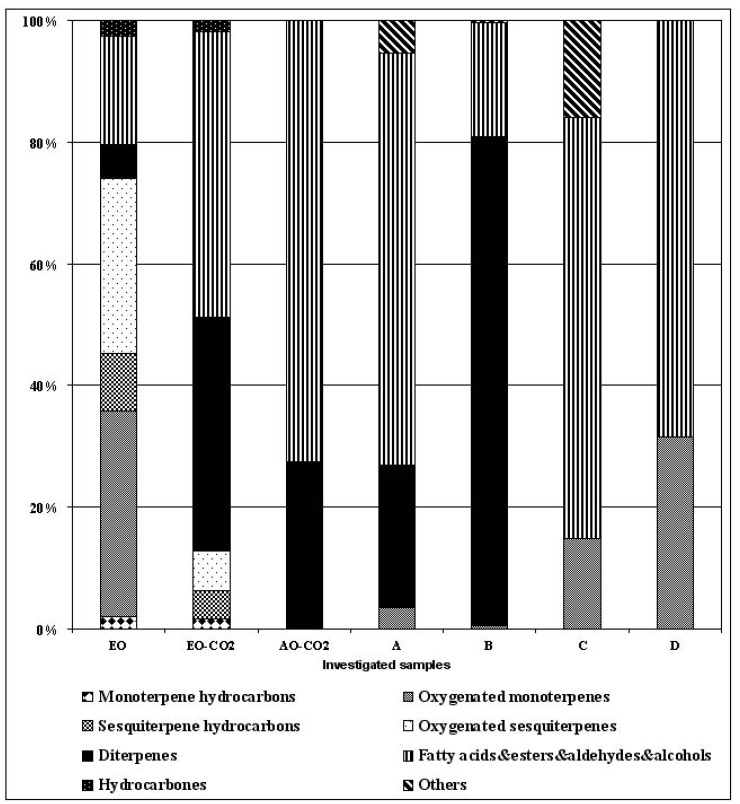
Comparative representation of particular groups of compounds (monoterpene hydrocarbons, oxygenated monoterpenes, sesquiterpene hydrocarbons, oxygenated sesquiterpene, diterpenes, fatty acids&esters&aldehydes&alcohols, hydrocarbons and others) in the investigated extracts obtained by hydrodistillation - HD (**EO**), supercritical carbon dioxide extraction - SC CO_2_ (**EO-CO_2_** and **AO-CO_2_**) and successive solvent extraction - SE (**A**, **B**, **C**, and **D**).

The content and chemical profile of the investigated supercritical and conventional S. *scardica* extracts (essential oil, **EO** obtained by HD, low volatile fraction, sample **EO-CO_2_**, obtained by supercritical fluid extraction at 10 MPa and 40 °C, the second fraction, sample **AO-CO_2_**, obtained by supercritical fluid extraction at 30 MPa and 40 °C, extracts **A**, **B**, **C** and **D**, obtained by successive SE were determined by GC and GC-MS analysis to establish the effect of the extraction method on the chemical composition (Table 1 and Figure 1) and on the antimicrobial activity of each extract (Table 2). 

Taking into account that the fraction A was obtained by SE using ethanol as non-selective solvent, we performed successive extraction of the A applying solvents with different polarity in order to enable the separation of the constituents regarding their polarity. Hence, we obtained extracts B (diethyl ether fraction), C (ethyl acetate fraction), and D (*n*-butanol fraction).

### 2.1. Chemical Composition of Investigated Samples

The constituents were analyzed by GC and GC-MS followed by calculation of Kovatz indices. In total, 133 compounds were identified (Table 1) in the investigated samples EO, EO-CO_2_, AO-CO_2_, A, B, C, and D accounting 89.12, 87.69, 98.12, 99.98, 90.30, 96.09 and 79.74% (respectively). 

In the EO sample, oxygenated monoterpenes were the major constituents, but with significant amount of oxygenated sesquiterpenes and fatty acids with their esters, as well (30.01, 25.54 and 15.96%, respectively). Although monoterpene hydrocarbons were previously reported as the main constituents of the essential oil of several *Sideritis* species, including *S. scardica* [21], in EO monoterpene hydrocarbons compounds represented only 1.83%. According to the previously published data, in the Macedonian *S. scardica* essential oil, the most abundant compound was α-cadinol, whereas in the oil of Bulgarian origin the main components were diterpenic compounds and octadecanol (over 20%) [22]. In our sample, diterpenes constituted a significant percentage; with octadecanol representing only 0.21% in oil. The most abundant compounds were hexadecanoic acid, myristicin, menthol, caryophyllene oxide, and τ-muurolol (12.92, 5.23, 4.90, 4.84, and 3.62%, respectively). 

Significant differences were established between the chemical profiles of the essential oils obtained by HD and SC CO_2_ applying the pressure of 10 MPa and temperature of 40 °C as extraction conditions (the samples EO and EO-CO_2_, respectively). Namely, fatty acids with their esters and diterpenes represented the main groups of the compounds in EO-CO_2_, with 41.16 and 33.75%, respectively (Table 1, Figure 1). The main components were hexadecanoic acid, dihydroxyl derivative of sandaracopimar-8(14),15-diene, (*E*)-ferruginol acetate and ethyl hexadecanoate (18.59, 14.89, 11.82 and 11.73%, respectively). 

The second fraction AO-CO_2_ obtained by SC CO_2_ extraction, maintaining the same temperature, but increasing the pressure to 30 MPa, was mainly characterized by the presence of fatty acids and diterpenes, as well, (71.07 and 26.81%, respectively). The major compounds were hexadecanoic and linoleic acids (43.22 and 24.80%, respectively), and diterpenes—the dihydroxy derivative of sandaracopimar-8(14),15-diene and (*E*)-ferruginol acetate (11.49 and 11.22% respectively). Besides, in all other investigated extracts fatty acids with their esters remained the major components, with the exception of fraction B, the extracts obtained by SE with diethyl ether as non-polar solvent, which was abundant in diterpenes, representing more than 70% of the chemical composition analyzed by GC-MS. The samples AO-CO_2_ and A had relatively similar patterns of the constituents fatty acids and diterpenes were the most abundant in A, as well, representing 67.75 and 23.3%, respectively. Hexadecanoic and dodecanoic acid (46.57 and 17.05%, respectively) and the dihydroxy derivative of sandaracopimar-8(14),15-diene, (*E*)-ferruginol acetate and (*Z*)-ferruginol acetate (10.79, 8.25 and 4.26%, respectively) diterpenes were the major components.

As stated above, fraction B contained mainly diterpenes, which were not detected in the extracts C and D. The main components in B were (*E*)-ferruginol acetate and the dihydroxy derivative of sandaracopimar-8(14),15-diene, (65.47 and 6.25%, respectively). Fractions C and D were the fractions abundant in fatty acids and their esters (66.48 and 54.59%, respectively), with differences in the type of fatty acids and their esters present in greatest quantity—as determined, hexadecanoic and dodecanoic acids were the major components in C (29.89 and 25.56%, respectively), whereas dodecanoic acid and decyl acetate (35.78 and 12.54%, respectively) were the most abundant in D. Besides, both samples C and D, contained the oxygenated monoterpene (*E*)-coniferyl alcohol in a significant percentage (11.81 and 18.69%, respectively), while the sample C contained cyclopentadecanolide (15.35%), as well. The comparative representation of the identified compounds in all investigated samples, classified in the different chemical groups, was typified in Figure 1.

Many studies have been performed on the chemical composition of essential oil from *Sideritis* species using the GC–MS and GC techniques. In spite of fact that the Lamiaceae family is well-known because of its essential oil content, *Sideritis* species cannot be considered rich in essential oil. Nevertheless a correlation between the oil yield and the main group of constituents has been established the higher the essential oil yield, the higher the monoterpene hydrogencarbon content. The large number of studies on essential oils composition in *Sideritis* can explain the polymorphism among the populations and the existence of new species, chemical varieties and hybrids. Several *Sideritis* essential oils are characterized by high contents of monoterpene hydrocarbons with α-pinene, β-pinene, sabinene, myrcene or limonene as the main compounds [21,22]. The presence of important sesquiterpene hydrocarbons, particularly δ-cadinene and β-caryophyllene, has been usually confirmed. Other essential oils are rich in oxygenated sesquiterpenes, such as α-cadinol, bisabolol or muurol-5-en-4β-ol as the main compounds, and finally diterpene compounds have been found in *Sideritis* essential oils. The presence of diterpenes as volatile compounds has been described in other genus such as *Cistus*, *Wollemia*, *Juniperus* and *Helichrysum*, characterized by what occurs in *Sideritis* with the presence of a large number of these compounds in the aerial part extracts. Turkish endemic species *S. bilgerana*, *S. ozturkii* and *S. cilicica* were rich in the monoterpene hydrocarbons α- and β-pinene. *S. cilicica* has been shown to have relatively high content of β-phellandrene [23]. In the group of *Sideritis* species rich in sesquiterpenes the main constituents have been found to be β-caryophyllene, D germacrene and calamene (*S. curvidens*, *S. montana*). Oxygenated derivatives are not common as main constituents in *Sideritis* species. Oxygenated monoterpenes, alongside with thymol, are characteristic consituents in *S. romana*. Oxygenated sesquiterpenes predominate in essential oils of *S. phlomoides* and *S. taurica*. The main constituents of *S. congesta* and *S. argyrea* essential oil were α- and β-pinene, while limonene was the major one in *S. perfoliata* essential oil. *S. condensata* provided an essential oil with high proportions of β-caryophyllene and α-pinene [24]. *S. perfoliata* and *S. dichotoma* essential oils are rich in diterpenes [25]. Monoterpene hydrocarbones has also been reported as main constituent in *Sideritis* species growing in Greece, and in some Spanish species, as well. In the essential oil of Spanish endemic species *S. ibanyezii*, sabinene and α-pinene have been found as main compounds [26,27]. The same monoterpene hydrocarbons, as well fenchone and cineole were the main constituents in the essential oil of *S. pusillafrom* the Iberian Peninsula [28].

According to presented data, the chemical composition of the analyzed samples revealed the existence of different patterns in comparison to the chemical profile of *S. scardica* already investigated by other authors [21,22]. Namely, diterpenes and fatty acids and their derivatives represented significant groups of compounds in our samples in contrast to others abundant in monoterpene hydrocarbons or oxygenated sesquiterpenes. Besides, in this work the investigated *S. scardica* essential oil obtained by hydrodistillation, contained oxygenated monoterpenes in the highest percentage.

### 2.2. Antimicrobial Activity

In this work, Gram-positive bacteria, *Streptococcus pyogenes*, *Streptococcus canis*, *Moraxella catarrhalis*, *Staphylococcus aureus*, methicillin resistant *Staphylococcus aureus*, *Corynebacterium pseudotuberculosis*, *Enterococcus faecalis*, Gram-negative bacteria *Escherichia coli*, *Pseudomonas aeruginosa*, *Klebsiella pneumoniae*, *Pasteurella multocida* and *Haemophilus sp*., and yeast *Candida albicans* were the tested microorganisms. *Pseudomonas aeruginosa* is a huge medical and veterinary problem with its intrinsic resistance to many antibiotics and disinfectants, and its ability to develop resistance to every so called “antipseudomonal antibiotic”, including carbapenems and ureidopenicillins [29,30]. *Candida albicans* infections are usually chronic and hard to treat, especially in children because of strong nephrotoxic and hepatotoxic side effects of some antifungals, especially ketoconazole [31,32]. On the other hand, staphylococci and streptococci, statistically are the most frequent cause of skin infections in humans and animals, whether in hospitals or in the community [32]. Unlike streptococci, which are usually susceptible to penicillins, staphylococci are hard to treat due to their ability of developing resistance to antibiotics and disinfectants [32,33]. At the same time, staphylococci are often the causative agents of secondary skin infections, usually after bites of insects or allergies, so, in all this cases antistaphylococcal therapy is needed [31]. The rest of the investigated strains only occasionally occur as causative agents of infections, mostly in immunocompromised patients.

Overall, minimal inhibitory concentration values (MIC values from 40 to ≥2,560 μg/mL) of the investigated extracts, presented in Table 2, indicated a strong to a moderate antibacterial activity of the investigated *S. scardica* extracts against the tested microorganisms. Investigated Gram-positive bacteria were more susceptible in comparison to investigated Gram-negative bacteria, with the exception of *Pasteurella multocida* and *Haemophilus sp.* Investigated extracts showed slight differences in their antimicrobial activity, but the common feature for all of them was the strongest activity against Gram-negative bacteria *Pasteurella multocida* and *Haemophilus sp*. and Gram-positive bacterium *Corynebacterium pseudotuberculosis* (MIC values 40–640 μg/mL). The strongest antibacterial activity was determined against *Haemophylus sp.* for the extracts C and D, with obtained MIC values of 40 and 80 µg/mL, respectively. The same extracts exhibited strong antimicrobial activity against *Corynebacterium pseudotuberculosis*, with MIC values of 80 µg/mL. Essential oil obtained by HD exhibited the strongest activity against *Corynebacterium pseudotuberculosis* and *Haemophylus sp.* with MIC values of 640 μg/mL, while moderate activity was determined against *Staphylococcus pyogenes*, *Moraxella catarrhalis* and *Pasteurella multocida*. Regarding the SC CO_2_ extracts (EO-CO_2_ and AO-CO_2_), practically all tested strains showed the same susceptibility; with the best results being obtained against *Corynebacterium pseudotuberculosis* (MIC value was 320 µg/mL) for the both extracts. All extracts obtained by SE (A, B, C, and D) demonstrated moderately strong or strong antibacterial activity against all investigated Gram-positive bacteria, with MIC values from 80 to 2,560 µg/mL. Interestingly, extracts C and D exhibited moderate activity against MRSA with MIC values 640 µg/mL. Investigated *Escherichia coli* and *Klebsiella pneumoniae* strains proved to be the most resistant to the applied concentration of the all investigated extracts, with MIC ≥ 2,560 µg/mL. All investigated extracts inhibited the growth of *Candida albicans* and *Pseudomonas aeruginosa* strains at a concentration at 2,560 µg/mL, with the exception of B and C (MIC ≥ 2,560 µg/mL).

There are several reports on the antimicrobial activity of *Sideritis* essential oils. The antimicrobial activity of *S. perfoliata* and *S. trojana* essential oils was tested against *Escherichia coli*, methicillin-resistant *Staphylococcus aureus*, *Enterobacter aerogenes*, *Salmonella typhimurium*, *Bacillus cereus*, *Staphylococcus epidermidis* and *Candida albicans*. The antimicrobial assay results indicated that *E. coli*, methicillin-resistant *S. aureus*, *E. aerogenes*, *B. cereus*, and *C. albicans* were moderately inhibited by the oil of *S. trojana*, but the oil showed strong inhibitory effects against *S. epidermidis*. *S. perfoliata* oil, on the other hand, was less active against the test microorganisms except for *C. albicans*. The occurrence of a higher content of oxygenated derivatives of mono- and sesquiterpenes (20%) in the oil of *S. trojana* may be responsible for the better antimicrobial activity [23,34]. In addition, there are several reports about the antimicrobial activity of essential oil from Spanish *Sideritis* species, *S. angustifolia*, *S. funkiana*, *S. javalambrensis*, *S. leucantha*, *S. mugronensis* and *S. tragoriganum* inhibited Gram-positive bacteria, *Staphylococcus aureus*, *Mycobacterium phlei* and the fungi *Candida albicans* growth, whereas they did not show any activity against Gram-negative bacteria. Similar results were achieved in the investigation of essential oil of *S. curvidens* and *S. lanata*, which had no effect against any Gram-negative bacteria, but with a significant activity on Gram-positive bacteria [35,36]. 

On the contrary, essential oils from *S. cilicica* and *S. bilgerana* exerted a significant inhibitory effect against several Gram-negative (*Salmonella typhimurium*, *Escherichia coli*) and Gram-positive (*Staphylococcus aureus*, *Bacillus cereus*, *Staphylococcus epidermidis*) bacteria, with a MIC value from 0.125 to 0.5 mg/mL, as well as against *Candida albicans* (MIC 0.03 mg/mL). This antibacterial activity could be due to the presence of α-pinene and β-pinene as the main constituents of both species [37]. Also, *S. italica* essential oil was investigated because of its antimicrobial activity which has been shown to be the higher against Gram-negative than Gram-positive bacteria, especially against *Pseudomonas aeruginosa* responsible for severe opportunistic infections and very often resistant to conventional antibiotics [1]. Besides, the shown strong inhibiting activity against *Helicobacter pylori* justified the ethnopharmacological use of *S. italica* as an antiulcer agent.

Not only the essential oil, but various *Sideritis* extracts possess significant antibacterial activity. According to the performed study by Sagdic *et al*. [38], the methanolic extracts of *S. ozturkii* and *S. caesarea*, had considerable antimicrobial activity. Linearol, foliol, epicandicandiol and siderol which were found in the mentioned *Sideritis* species were investigated for antibacterial activities as well and epicandicandiol had the highest antimicrobial activity against *E. coli*. The acetone and methanol extracts of *S. tmolea* P. H. Davis were tested against standard bacterial strains. As the result of the activity studies, it is found that *Sideritis* species crude acetone and methanol extracts have not shown considerable antimicrobial or antituberculous activity [39], but the inhibition of clotrimmazole-resistant *C. albicans* by some *Sideritis* species from Turkey were reported [40]. As well, *in vitro* studies indicated that a series of *ent*-manoyl oxides from *S. varoi* and their synthetically obtained derivatives inhibited the growth of *Leishmania donovani* [41].

The observed antimicrobial activity of the investigated extracts in this work might be attributed to the presence different types of terpenoids. Besides the established antimicrobial potential of monoterpenes, diterpenes have attracted considerable attention recently. Namely, diterpenes (especially pimarane type) have been reported to display important biological activities, including antimicrobial activity [42]. Also, the antimicrobial activity might be the results of the various compounds present in the investigated extracts, but not identified by the applied analysis techniques.

There are no data on referential MIC values of plant extracts upon which categorization of investigated microorganisms could be done (susceptible or resistant) and antimicrobial potency of plant extracts is often estimated by comparing MIC values of plant extracts to MIC values of antibiotics. The scientific basis of such practice is unclear and microbiologically and pharmacologically this is the wrong principle because of different pharmacokinetics and metabolism of plant extract and antibiotics. In other words, higher MIC values of plant extracts do not necessarily mean weak antimicrobial potency [43,44]. For the purpose of the determination of MIC values of antibiotics or plant extracts, investigated substances first have to be diluted in order to find the lowest concentration in which they demonstrate antimicrobial activity [45,46,47]. Regarding this, in this research, extracts were previously diluted as described in Experimental. Dilution has been done by adding 25.6 µL (0.0256 mL) of investigated sample in 1 mL of DMSO (with density correction for every extract). The measured volume of investigated extracts was very small and from the microbiological point of a view, MIC values of 1,280 µg/mL and 2,560 µg/mL might be interpreted as no or weak antimicrobial activity [43,48]. Contrary to that, according to Aligiannis *et al*. [26], the antimicrobial activity of investigated essential oils of *S. sipyle*, *S. clandestine* and *S. raeseri* was characterized as strong or moderate, with MIC values ranging from 650 to 9,900 µg/mL, being significantly higher in comparison to those observed in this work.

### 2.3. The Extraction Yields 

The yields of the performed extractions are presented in the Table 3. The results were as expected, according to the low selectivity of the polar solvent used for obtaining the sample A.

### 2.4. The Kinetics of SC-CO_2_ Extraction

**Figure 2:**
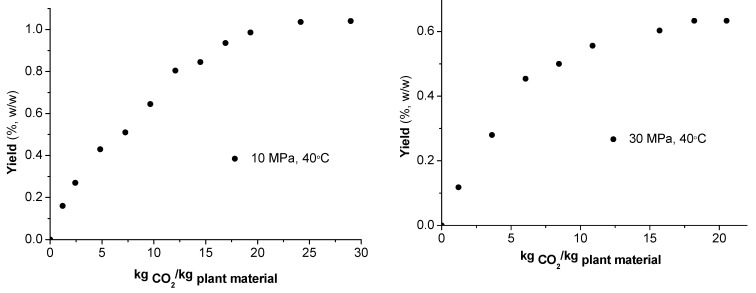
Experimental results for supercritical fluid extraction from *S. scardica* at 10 MPa and 40 °C (left), and 30 MPa and 40 °C (right).

The kinetics of the extraction processes are depicted in Figure 2. The yield is shown as a function of the specific SC CO_2_ consumption. The results indicate that the total CO_2_ consumption was 1,200 g for the first, and 870 g for the second fraction extraction. The kinetics of both experiments was obviously similar. However, the first fraction consists mainly of low volatile components that represent essential oil, while the second fraction (defined as nonvolatile) consists mainly of components characterized with higher molecular weight.

## 3. Experimental 

### 3.1. Plant Material

Wild growing species *Sideritis scardica* Griseb., Lamiaceae were collected on Shara Mountain (at the foothills of the Ljuboten, at ca. 1300 m) during the time of flowering. Plant material was air dried, packed in paper bags and kept in a dark and cool place until analysis. Plant material was verified and the voucher specimen of the plant (SS/08) was deposited at Herbarium of Botanical Garden, Jevremovac, Belgrade, Serbia. The plant material was milled in a blender for 60 s and immediately subjected to hydrodistillation (HD) or supercritical CO_2_ extraction (SC CO_2_), and solvent extraction, as well. The average particle size of milled herbs was 0.40 mm (used for all performed extractions).

### 3.2. Essential Oil Extraction by Hydrodistillation

Dried, milled herb (50 g) of *S. scardica* was distilled using 700 mL distilled water according to the standard Clevenger method (4 h) and a yellow viscous volatile essential oil with a balsamic odor was collected. The obtained essential oil (Sample *EO*) was kept in a sealed vial at 4 °C. The yield (w/w) of essential oil was 0.03% (on a dry weight basis).

### 3.3. Supercritical Fluid Extraction 

**Figure 3:**
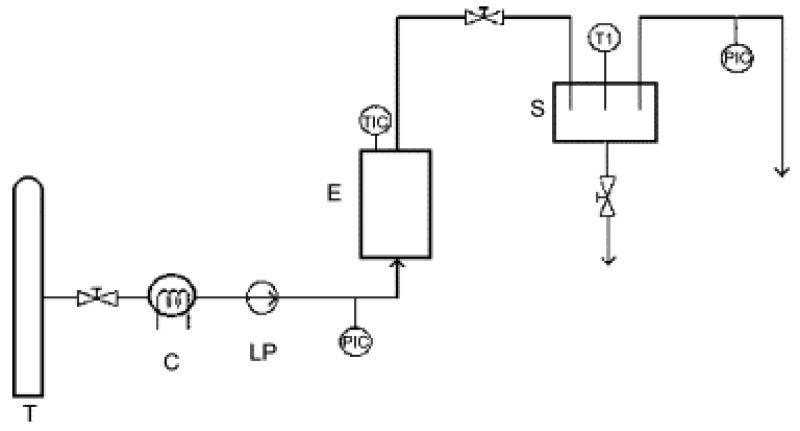
Schematic presentation of the autoclave engineers screening system—T: CO_2_ storage tank; C: cryostat; LP: high pressure liquid pump; E: extractor vessel; S: separator vessel.

Extractions with supercritical carbon dioxide (SC CO_2_) were performed on a laboratory scale equipment, in an Autoclave Engineers SCE Screening System with a 150 cm^3^ extractor vessel previously described [49] and shown in Figure 3. 

Plant material (41.4 g) was milled and sieved. The fraction with an average particle diameter of 0.4 mm (collected between sieves of 0.2 mm and 0.6 mm) was used for the experiments. Supercritical extractions with carbon dioxide were performed fractionally. The pressure and temperature conditions for the extraction of the first fraction were 10 MPa and 40 °C respectively, while the SC CO_2_ flow rate was 0.67 kg/h (obtained sample **EO-CO_2_**). After the plant material was exhausted, the pressure was raised to 30 MPa and the extraction of the second fraction followed. The SC CO_2_ flow rate was 0.32 kg/h (obtained sample **AO-CO_2_**). Commercial carbon dioxide (99% purity) supplied by Tehnogas (Messer-Tehnogas, Serbia) was used for SC CO_2_, and dichloromethane and alcohol (GC purity, Sigma–Aldrich, Germany) was used for dissolution of supercritical extracts prior to GC-FID-MS analyses.

### 3.4. Solvent Extraction (SE)

The shade-dried and powdered aerial parts of *S. scardica* (200 g) were coarsely extracted using 70% (v/v) ethanol. The crude ethanol extract (**A**) was re-dissolved in distilled water, shaken vigorously and successively extracted with 200 mL of diethyl ether, 200 mL ethyl acetate, and 200 mL saturated *n*-butanol in a separating funnel,. The obtained extracts were labelled as the diethyl ether extract, **B** (0.9 g) ethyl acetate extract, **C** (0.4 g). and *n*-butanol extract, **D** (1.5 g), respectively. 

### 3.5. Gas Chromatography (GC-FID)

Gas chromatography analysis of the extracts was carried out on a HP-5890 Series II GC apparatus [Hewlett-Packard, Waldbronn (Germany)], equipped with a split–splitless injector and automatic liquid sampler, attached to a HP-5 column (25 m × 0.32 mm, 0.52 μm film thickness) and fitted with a flame ionization detector (FID). Carrier gas flow rate (H2) was 1 mL/min, split ratio 1:30, injector temperature was 250 °C, detector temperature 300 °C, while column temperature was linearly programmed from 40 to 260 °C (at rate of 4 °C /min), and then kept isothermally at 260 °C for 10 min. Solutions of samples in dichloromethane or alcohol were consecutively injected in amount of 1 μL. Area percent reports, obtained as result of standard processing of chromatograms, were used as base for the quantification analysis.

### 3.6. Gas Chromatography/Mass Spectrometry (GC-MS) 

The same analytical conditions as those mentioned for GC-FID were employed for GC/MS analysis, along with a column HP-5MS (30 m × 0.25 mm, 0.25 μm film thickness), using a HP G 1800C Series II GCD system [Hewlett-Packard, Palo Alto, CA, USA]. Helium was used as carrier gas. The transfer line was heated at 260 °C. Mass spectra were acquired in EI mode (70 eV); in the 40–450 *m/z* range. An amount of 0.2 μL of sample solution in dichloromethane or alcohol was injected. The components of the oil were identified by comparison of their mass spectra to those from the Wiley 275 and NIST/NBS libraries, using different search engines. Identification of the compounds was achieved by comparing their retention indices and mass spectra with those found in the literature [50] and supplemented by the Automated Mass Spectral Deconvolution and Identification System software (AMDIS ver. 2.1), GC-MS Librairies [51]. The experimental values for retention indices were determined by the use of calibrated Automated Mass Spectral Deconvolution and Identification System Software (AMDIS ver. 2.1), GC-MS Libraries [51], compared to those from available literature (Adams 2007) [50] and used as additional tool to confirm the MS findings. The relative proportion of the essential oil constituents were expressed as percentages obtained by peak area normaliyation, all relative response factors being taking as one.

### 3.7. In Vitro Antimicrobial Activity 

The investigation of the antibacterial activity of investigated samples **EO**, **EO-CO_2_**, **AO-CO_2_**, **A**, **B**, **C**, and **D** was performed on Gram-positive and Gram-negative bacterial species. From the group of Gram positive microorganisms, *Streptococcus pyogenes*, *Streptococcus canis*, *Moraxella catarrhalis*, *Staphylococcus aureus*, *Corynebacterium pseudotuberculosis*, and *Enterococcus faecalis* strains were chosen. From the group of Gram-negatives, *Klebsiella pneumoniae*, *Pseudomonas aeruginosa*, *Escherichia coli*, *Pasteurella multocida* and *Haemophilus* strains were selected. Pathogenic yeasts was also included in the investigation and a *Candida albicans* strain was chosen for that purpose. The investigated strains were isolated from skin and tonsils swabs taken from diseased persons and animals with infection symptoms, except *Staphylococcus aureus* ATCC 25923 and the methicillin-resistant *Staphylococcus aureus* referential strains (MRSA ATCC 43300) which were purchased from Becton Dickinson, USA. The isolation was made from clinical material delivered to the Microbiology Department, Faculty of Veterinary Medicine, Belgrade University. 

Conventional microbiological methods were applied for the purpose of isolation and identification and Columbia sheep blood agar (bioMerieux), MacConkey agar (bioMerieux), CNA agar with colistin and nalidixic acid (Becton Dickinson) and nutrient broth (BioLab) were used. For the isolation of *Candida albicans*, Sabouraud dextrose agar was used (BioLife). Identification of isolated strains was performed with BBL Crystal Gram-positive ID kit, BBL Crystal enteric/nonfermenter ID kit (Becton Dickinson), API 32 STAPH, API 20 NE and API 20 C AUX (bioMerieux). 

For the investigation of antibacterial activity and the determination of MIC values of the investigated samples, broth microdillution method was applied in accordance with the CLSI prescriptions for antimicrobial susceptibility testing [45,46,47]. For that purpose, Cation adjusted Mueller Hinton II broth was used (CAMHB, Becton Dickinson) with the addition of 1.6% bromcresol purple (Merck) in final concentration at 0.2 mL/200 mL for Gram-positives and 1% phenol red (Merck) at 1 mL/200 mL for Gram-negatives. Bromcresol purple and phenol red were added to obtain bacterial growth visibility. Sabouraud dextrose broth (BioLife) was used for yeasts with no indicators added. For streptococci, foetal bovine serum (Sigma) was added in CAMHB at final concentration at 5%. Dimethyl sulfoxide, (DMSO, Merck) was used as solvent for investigated samples. Investigated concentrations of investigated samples were 2560, 1280, 640, 320, 160, 80, 40, 20, 10, 5, 2,5 and 1,25 expressed in μg/mL. The samples were dissolved in DMSO at 25.600 μg/mL, then 1:10 dilution with CAMHB was made. Titration until desired concentrations was performed in microplate wells as previously described [45,46,47]. The final bacterial inoculum density of 5 × 10^5^ CFU/mL was achieved by adding 5 μL of 1–2 × 10^7^ CFU/mL suspension of investigated strain in microplate wells with 100 μL of previously added CAMHB. Microplates were incubated 18–24 h on 37 °C. For MIC values the broth with lowest oil concentration, with no visible bacterial growth, was used. 

## 4. Conclusions

The overall aim of this study was to contribute to the global search for bioactive natural products and convenient methods for their extraction. Hydrodistillation and solvent extraction are traditional techniques to recover compounds from aromatic plants. As an alternative method, supercritical carbon dioxide extraction was used and proved to be suitable for obtaining different plant extracts. The extraction method influenced the yield of the extraction and chemical composition performed by GC and GC-MS techniques of the investigated *S. scardica* extracts. The chemical profiles of the investigated extracts by GC-MS analysis revealed differences regarding the content of different compounds group - monoterpene and sesquiterpene hydrocarbons, oxygenated monoterpenes and sesquiterpenes, diterpenes, and fatty acids and their esters, as well. As observed differences in chemical compositions of investigated extracts were significant, the alternative SC CO_2_ extractions could not replace the conventional ones, regardless of the better yields of the extraction. Considering the literature, there were no data regarding the chemical composition of SC-CO_2_ extracts isolated from *S. scardica* at 10 MPa and 30 MPa at 40 °C, nor for the antimicrobial activity of *S. scardica* extracts or essential oil. The antimicrobial activity was detected at comparable levels for all investigated extracts, with obtained MIC values of 40–2,560 μg/mL. The lowest MIC values were detected for the extracts obtained by solvent extraction. The extracts obtained by supercritical carbon dioxide extraction exhibited more or the same activity against almost all investigated microorganisms in comparison to essential oil obtained by hydrodestilliation. In spite of the differences in the methods applied for the extraction, and the chemical composition of the investigated *S. scardica* extracts, as well, antimicrobial activity was not significantly influenced, revealing the possibility that the combination of diterpenes and fatty acids and their derivatives might be, at least, partly responsible for the shown activity, but the presence of other compound not identified by applied techniques should not be ignored.

## Figures and Tables

**Table 1:** Chemical composition of the supercritical (**EO-CO_2_**, **AO-CO_2_**) and conventional (**EO**, **A**, **B**, **C**, and **D**) *S. scardica* investigated extracts.

No	Extraction mode		HD	SC CO_2_		SE			
	Compound	K.I. ^a^	EO	EO-CO_2_	AO-CO_2_	A	B	C	D
1	α-thujene	925.5	0.30	-	-	-	-	-	-
2	thuja-2,4(10)-diene	953.0	-	1.33	-	-	-	-	-
3	sabinene	967.3	0.05	-	-	-	-	-	-
4	myrcene	967.4	1.25	0.24	-	-	-	-	-
5	*n*-Decane	985.5	0.07	-	-	-	-	-	-
6	β-phellandrene/limonene	1021.7	0.05	-	-	-	-	-	-
7	1,8-cineole	1023.2	0.08	-	-	-	-	-	-
8	γ-terpinene	1052.8	0.11	-	-	-	-	-	-
9	isobutyl acetoacetate	1084.9	0.31	-	-	-	-	-	-
10	linalool	1095.3	1.53	-	-	-	-	-	-
11	β-thujone	1097.8	0.45	-	-	-	-	-	-
12	α-thujone	1098.7	0.56	-	-	-	-	-	-
13	*trans-*pinocarveol	1130.8	0.34	-	-	-	-	-	-
14	camphor	1134.8	0.88	-	-	-	-	-	-
15	*trans-*verbenol	1138.8	t	-	-	-	-	-	-
16	iso-menthone	1145.8	1.75	-	-	-	-	-	-
17	borneol	1157.7	1.46	-	-	-	-	-	-
18	**menthol**	**1165.6**	**4.90**	-	-	-	-	-	-
19	terpinen-4-ol	1169.8	0.42	-	-	-	-	-	-
20	α-terpineol	1186.2	0.27	-	-	-	-	-	-
21	myrtenal	1190.8	0.42	-	-	-	-	-	-
22	myrtenol	1191.2	0.64	-	-	-	-	-	-
23	*trans-*dihydrocarvone	1199.5	0.07	-	-	-	-	-	-
24	β-cyclocitral	1214.0	0.09	-	-	-	-	-	-
25	neoiso-dihydrocarveol	1224.5	0.08	-	-	-	-	-	-
26	thymol methyl ether	1228.4	0.19	-	-	-	-	-	-
27	pulegone	1233.4	0.80	-	-	-	-	-	-
28	carvacrol methyl ether	1235.5	0.36	-	-	-	-	-	-
29	d-carvone	1242.1	0.37	-	-	-	-	-	-
30	piperotone	1250.7	0.27	-	-	-	-	-	-
31	**(*Z*)-chrysanthenyl acetate**	**1257.8**	-	-	-	-	-	**1.63**	**4.58**
32	isobornyl acetate	1277.5	1.10	-	-	-	-	-	-
33	(*E*)-anethole	1281.4	2.89	-	-	-	-	-	-
34	menthyl acetate	1286.4		-	-	-	-	-	-
35	**thymol**	**1291.1**	**1.97**	-	-	-	-	-	-
36	**carvacrol**	**1300.5**	**2.05**	-	-	**1.94**	**0.48**	**0.82**	**1.88**
37	(*E*)-dimetoxy citral	1341.0	-	-	-	1.62	-	-	-
38	3’-metoxy-acetophenone	1343.0	0.02	-	-	-	-	-	-
39	α-cubebene	1345.0	0.02	-	0.05	-	-	-	-
40	α-terpenyl acetate	1342.3	0.03	-	-	-	-	-	-
41	**decanoic acid**	**1364.0**	-	-	-	-	-	**0.10**	**5.47**
42	α-copeane	1365.6	0.11	0.34	-	-	-	-	-
43	β-bourbonene	1374.3	0.04	-	-	-	-	-	-
44	*trans*-β-demascenone	1376.9	0.21	-	-	-	-	-	-
45	**decyl acetate**	**1407.0**	-	-	-	-	-	**2.66**	**12.54**
46	α-dihydroionone	1389.0	0.27	-	-	-	-	-	-
47	β-funebrene	1395.2	0.30	-	-	-	-	-	-
48	*trans*-β-caryophyllene	1408.0	0.60	1.39	-	-	-	-	-
49	2,5-dimethyl-*p*-cymene	1417.4	0.07	-	-	-	-	-	-
50	*trans*-α-bergamotene	1425.9	0.06	-	-	-	-	-	-
51	α-humulene	1442.4	0.10	-	-	-	-	-	-
52	(*E*)-β-farnesene	1449.2	0.21	-	-	-	-	-	-
53	(*2E*)-dodecanal	1464.0	0.10	-	-	-	-	-	-
54	germacrene D	1470.3	0.52	-	-	-	-	-	-
55	(*E*)-β-ionone	1478.0	1.15	-	-	-	-	-	-
56	(*E*)-muurola-4(14),5-diene	1482.2	0.03	-	-	-	-	-	-
57	valencene	1484.8	0.35	-	-	-	-	-	-
58	α-muurolene	1490.2	0.33	-	-	-	-	-	-
59	β-bisabolene	1499.3	0.19	0.41	-	-	-	-	-
60	χ-cadinene	1503.1	0.13	0.89	-	-	-	-	-
61	7-epi-α-selinene	1520.0	0.66	0.94	-	-	-	-	-
62	(*E*)-calamenene	1521.0	0.36	-	-	-	-	-	-
63	**myristicin**	**1522.0**	**5.23**	-	-	-	-	-	-
64	δ-cadinene	1522.0	0.10	-	-	-	-	-	-
65	ether-italicane	1531.1	t	-	-	-	-	-	-
66	α-calacorene	1532.7	1.31	-	-	-	-	-	-
67	β-calacorene	1553.3	1.21	-	-	-	-	-	-
68	(*E*)-nerolidol	1557.1	0.06	-	-	-	-	-	-
69	**dodecanoic acid**	**1565.0**	-	-	-	**17.05**	**6.96**	**25.56**	**35.78**
70	spathulenol	1577.0	1.97	-	-	-	-	-	-
71	**caryophyllene oxide**	**1582.0**	**4.84**	**2.44**	**0.07**	-	-	-	-
72	viridiflorol	1592.0	1.23	-	-	-	-	-	-
73	carotol	1593.5	t	-	-	-	-	-	-
74	ledol	1594.3	0.99	-	-	-	-	-	-
75	diepi-α-cedrenepoxide	1607.0	t	-	-	-	-	-	-
76	humulene epoxide II	1608.0	0.56	-	-	-	-	-	-
77	ledene	1613.0	0.32	-	-	-	-	-	-
78	(*E*)-isolongifolanene	1618.8	0.56	-	-	-	-	-	-
79	α-colocalene	1622.0	0.14	-	-	-	-	-	-
80	muurola-4,10(14)-dien-1-β-ol	1630.0	0.61	0.21	-	-	-	-	-
81	caryophylla-4(12),8(13)-dien-5-β-ol	1639.0	0.51	-	-	-	-	-	-
82	**τ-muurolol**	**1640.6**	**3.62**	-	-	-	-	-	-
83	α-muurolol	1645.7	0.19	-	-	-	-	-	-
84	α-selin-11-en-4-ol	1658.1	1.65	-	-	-	-	-	-
85	(*E*)-calamenen-10-ol	1668.2	1.45	-	-	-	-	-	-
86	**valeranone**	**1674.4**	**2.15**	**1.06**	-	-	-	-	-
87	cadelene	1675.0	1.51	-	-	-	-	-	-
88	α-germacra-4(15),5,10(14)-trien-1-ol	1685.3	1.21	-	-	-	-	-	-
89	α-bisabolol	1685.7	0.32	0.27	-	-	-	-	-
90	acorenone	1692.0	0.26	-	-	-	-	-	-
91	**2-(*E*)-tridecanol acetate**	**1703.0**	**2.50**	**0.65**	**0.19**	-	-	-	-
92	**(*E*)-coniferyl alcohol**	**1735.6**	-	-	-	-	-	**11.81**	**18.69**
93	benzyl benzoate	1761.8	0.02	-	-	-	-	-	-
94	β-bisabolenal	1768.9	0.22	-	-	-	-	-	-
95	β-bisabolenol	1786.1	1.40	0.53	-	-	-	-	-
96	(*2Z,6E)-*farnesyl acetate	1821.0	-	0.44	-	-	-	-	-
97	**cyclopentadecanolide**	**1826.6**	-	-	-	**4.01**	**0.34**	**15.35**	-
98	(*Z*)-lanceol acetate	1858.0	-	0.66	-	-	-	-	-
99	hexadecanol	1878.8	-	-	-	-	-	0.63	0.42
100	(*5E,9E*)-farnesyl acetone	1907.9	0.45	-	0.12	-	-	-	-
101	methyl hexadecanoate	1922.0	-	-	0.27	-	-	-	-
102	*ent*-rosa-5,15-diene	1933.9	0.15	-	-	-	-	-	-
103	**pimaradiene**	**1948.8**	**0.05**	**1.52**	**2.84**	-	-	-	-
104	**hexadecanoic acid**	**1966.6**	**12.92**	**18.59**	**43.22**	**46.57**	**8.53**	**29.89**	**0.38**
105	**ethyl hexadecanoate**	**1992.0**	**0.20**	**11.82**	-	**4.13**	**1.23**	**7.64**	-
106	kaur-15-ene	1997.0	1.88	0.55	-	-	-	-	-
107	13-epi-manool oxide	2009.9	0.62	-	-	-	-	-	-
108	manool	2041.7	0.54	-	-	-	-	-	-
109	13-epi-manool	2059.0	0.53	-	-	-	-	-	-
110	**octadecanol**	**2077.0**	**0.21**	**6.20**	**1.84**	**t**	**t**	-	-
111	methyl linoleate	2095.0	0.01	1.34	0.11	-	-	-	-
112	methyl oleate	2104.0	-	-	0.18	t	-	-	-
113	**linoleic acid**	**2132.0**	**0.12**	**0.71**	**24.80**	-	**t**	-	-
114	oleic acid	2141.0	-	0.80	-	-	-	-	-
115	phytol acetate	2170.6	-	-	0.46	-	-	-	-
116	ugandensodial	2190.0	-	-	-	1.36	-	-	-
117	7α-hydroxy manool	2237.0	0.41	0.61	-	-	-	-	-
118	3β-sandaracopimardienol	2269.0	0.20	-	-	t	t	-	-
119	sandaracopimarinol	2269.0	0.37	1.26	1.26	-	-	-	-
120	tricosane	2300.0	0.38	0.40	-	-	t	-	-
121	isopimarol	2310.4	0.22	1.49	-	-	-	-	-
122	** (*E*) -ferruginol acetate**	**2357.0**	-	**11.73**	**11.22**	**8.25**	**65.47**	-	-
123	methyl strictate	2387.0	-	1.70	-	-	-	-	-
124	9-octadecen-1-ol	2396.4	-	1.05	-	-	-	-	-
125	** (*Z*) -ferruginol acetate**	**2406.0**	-	-	-	4.26	1.04	-	-
126	pentacosane	2486.0	0.41	1.23	-	-	-	-	-
127	**dihydroxysandaracopimar-8(14),15-diene ^b^**	**2506.0**	-	**14.89**	**11.49**	**10.79**	**6.25**	-	-
128	hexacosane	2600	-	t	-	-	-	-	-
129	heptacosane	2700.0	1.28	t	t	-	-	-	-
130	octacosane	2800.0	-	t	t	-	-	-	-
131	nonacosane	2900.0	-	t	-	-	-	-	-
132	triacontane	3000.0	0.08	-	-	-	-	-	-
133	dotriacontane	3200.0	0.01	-	-	-	-	-	-
**Total**			**89.12**	**87.69**	**98.12**	**99.98**	**90.30**	**96.09**	**79.74**
*Monoterpene hydrocarbons*			1.83	1.57	-	-	-	-	-
*Oxygenated monoterpenes*			**30.01**	-	-	3.56	0.48	**14.26**	**25.15**
*Sesquiterpene hydrocarbons*			8.63	3.97	0.05	-	-	-	-
*Oxygenated sesquiterpenes*			**25.54**	5.61	0.19	-	-	-	-
*Diterpenes*			4.97	**33.75**	**26.81**	**23.30**	**72.76**	-	-
*Fatty acids&esters&aldehydes&alcohols*			**15.96**	**41.16**	**71.07**	**67.75**	**16.72**	**66.48**	**54.59**
*Hydrocarbones*			2.16	1.63	t	-	t	-	-
*Others*			0.02	-	-	5..37	0.34	**15.35**	-

^a^ Kovats index; t = trace (percentage less than 0.01%); ^b^ MW 304, the peaks at m/z 121 and 133 supports a sandaracopimara-8(14),15-diene diterpene structure and the fragments 286[M − H2O]^+^ (100), 268 (30) indicate hydroxyl groups; Position of OH groups not determined.

**Table 2:** Results of testing the antibacterial activity on Gram-positive, Gram-negative bacteria and yeast of the supercritical (**EO-CO_2_**, **AO-CO_2_**) and conventional (**EO**, **A**, **B**, **C**, and **D**) *S. scardica* investigated extracts.

Strain	MIC VALUES µg/mL							
	EO	EO-CO_2_	AO-CO_2_	A	B	C	D	Gentamicin
***Gram-positive bacteria***								
*Streptococcus pyogenes* 1, tonsils swab	1280	640	640	1280	640	1280	1280	≤4
*Streptococcus pyogenes* 2, tonsils swab	1280	640	640	1280	1280	1280	2560	≤4
*Streptococcus canis*, tonsils swab dog	2560	2560	2560	2560	2560	2560	>2560	≤4
*Moraxella catarrhalis*, tonsils swab	1280	2560	2560	1280	1280	1280	2560	≤4
*Staphylococcus aureus*, ATCC 25923	>2560	>2560	>2560	1280	1280	1280	1280	≤4
*Staphylococcus aureus*, CI, tonsils swab	>2560	2560	2560	1280	1280	640	1280	≤4
MRSA ATCC 43300	>2560	2560	2560	1280	1280	640	640	≤4
*Corynebacterium pseudotuberculosis*, tonsils swab	640	320	320	160	320	80	80	≤4
*Enterococcus faecalis*, tonsils swab	2560	2560	2560	>2560	>2560	>2560	1280	≤4
								
***Gram-negative bacteria***								
*Escherichia coli*, ATCC 25922	2560	>2560	>2560	2560	>2560	>2560	2560	≤4
*Escherichia coli*, CI, skin swab	>2560	>2560	>2560	2560	>2560	>2560	2560	≤4
*Pseudomonas aeruginosa*, tonsils swab	2560	2560	2560	2560	>2560	>2560	2560	≤4
*Klebsiella pneumoniae*, tonsils swab	>2560	>2560	>2560	2560	>2560	>2560	2560	≤4
*Pasteurella multocida* tonsils swab, dog	1280	1280	1280	640	640	320	320	≤4
*Haemophilus sp*., nose swab	640	640	640	320	320	40	80	≤4
								
***Yeast***								
*Candida albicans*, tonsils swab	2560	2560	2560	2560	>2560	>2560	2560	-

All swabs were taken from humans, except were indicated. CI - clinical isolates.

**Table 3:** The yields, calculated as the amount of extract compared to the total mass of solid material at the beginning of the extraction process for the supercritical (**EO-CO_2_**, **AO-CO_2_**) and conventional (**EO**, **A**, **B**, **C**, and **D**) *S. scardica* investigated extracts.

Investigated samples	Yield of extraction (%)
**EO**	0.03
**EO-CO_2_**	1.04
**AO-CO_2_**	0.63
**A**	16.70
**B**	0.50
**C**	0.20
**D**	0.70
